# Molecular and Biochemical Analysis of Chalcone Synthase from *Freesia hybrid* in Flavonoid Biosynthetic Pathway

**DOI:** 10.1371/journal.pone.0119054

**Published:** 2015-03-05

**Authors:** Wei Sun, Xiangyu Meng, Lingjie Liang, Wangshu Jiang, Yafei Huang, Jing He, Haiyan Hu, Jonas Almqvist, Xiang Gao, Li Wang

**Affiliations:** 1 Institute of Genetics and Cytology, Northeast Normal University, Changchun, China; 2 Department of Cell and Molecular Biology, Uppsala University, Uppsala Biomedical Center, Uppsala, 596, S-75124, Sweden; Agriculture Canada, CANADA

## Abstract

Chalcone synthase (CHS) catalyzes the first committed step in the flavonoid biosynthetic pathway. In this study, the cDNA (*FhCHS1*) encoding CHS from *Freesia hybrida* was successfully isolated and analyzed. Multiple sequence alignments showed that both the conserved CHS active site residues and CHS signature sequence were found in the deduced amino acid sequence of *FhCHS1*. Meanwhile, crystallographic analysis revealed that protein structure of FhCHS1 is highly similar to that of alfalfa CHS2, and the biochemical analysis results indicated that it has an enzymatic role in naringenin biosynthesis. Moreover, quantitative real-time PCR was performed to detect the transcript levels of *FhCHS1* in flowers and different tissues, and patterns of *FhCHS1* expression in flowers showed significant correlation to the accumulation patterns of anthocyanin during flower development. To further characterize the functionality of *FhCHS1*, its ectopic expression in *Arabidopsis thaliana tt4* mutants and *Petunia hybrida* was performed. The results showed that overexpression of *FhCHS1* in *tt4* mutants fully restored the pigmentation phenotype of the seed coats, cotyledons and hypocotyls, while transgenic petunia expressing *FhCHS1* showed flower color alteration from white to pink. In summary, these results suggest that *FhCHS1* plays an essential role in the biosynthesis of flavonoid in *Freesia hybrida* and may be used to modify the components of flavonoids in other plants.

## Introduction

The flower color, which is mainly determined by betalains, carotenoids and flavonoids, is one of the most important characteristics in ornamental plants [1,2]. Betalains, nitrogen-containing compounds derived from tyrosine, confer violet to red and yellow to orange coloration to flowers but are found exclusively in *Caryophyllales* [3,4]. Carotenoids furnish flowers with different colors ranging from yellow to red and are essential for photosynthesis. Unlike betalains and carotenoids, flavonoids are often present in the same plant tissues to create co-pigmentation phenomenon, and this combination increases the color variety [5,6]. In addition, flavonoids are the most abundant and widely distributed pigments in the plant kingdom, and they are largely responsible for diverse pigmentation from shiny orange to pink, red, violet and blue colors in the flowers and fruits. So far, more than 6,000 flavonoids have been identified [7]. Flavonoids are one of the most important secondary metabolites that comprise several different classes of compounds such as chalcones, flavones, flavonols and anthocyanins, and have a wide variety of biological functions, including protection of cells against UV radiation, resistances against phytopathogens and herbivores, signal molecules in plants and microbes interaction, pollen growth and development, root nodule organogenesis, auxin transport, and most importantly, they pigment flower to attract pollinators and disperse the fruits and seeds [8–11]. It has been suggested that flavonoids may have played a crucial role in the early evolution of land plants, first as chemical messengers and then as UV screeners [12,13]. Meanwhile, it has been reported that flavonoids also have a close connection with human health. Besides the remarkable anti-oxidant, anti-virus and anti-proliferative biological activities, they were also applied for preventative maintenance on atherosclerotic arteries and treatment of cardiovascular disease as well as many other diseases [14–16].

Over the past few decades, the flavonoid biosynthetic pathway has been well established by using petunia (*Petunia hybrida*), snapdragon (*Anfirrhinum majus*), *Arabidopsis* (*Arabidopsis thaliana*) and maize (*Zea mays*) as models [17,18]. Briefly, the biosynthesis of flavonoid starts with the condensation of one molecule of *p*-coumaroyl-CoA (derived from the general phenylpropanoid pathway) with three molecules of malonyl-CoA, resulting in naringenin chalcone and this reaction is conducted by chalcone synthase (CHS). Then the naringenin chalcone is rapidly isomerized to naringenin by chalcone isomerase (CHI). From these central intermediates, the biosynthetic pathway diverges into side branches, each yielding a different class of flavonoids. Subsequently, flavanone 3-hydroxylase (F3H) catalyzes the hydroxylation of naringenin (the C-ring) to produce dihydrokaempferol, which is further hydroxylated at the B-ring to form dihydroquercetin and dihydromyricetin. Finally, the dihydroflavonols are converted into colored anthocyanins by the actions of dihydroflavonol reductase (DFR), anthocyanidin synthase (ANS) and flavonoid 3-*O*-glucosyltransferase (3GT) [19–21]. Furthermore, the action of flavonol synthase (FLS), leucoanthocyanidin reductase (LAR) and anthocyanidin reductase (ANR) leads to branching of the flavonoid pathway to flavonols and proanthocyanidins, respectively [22–24]. In conclusion, CHS is the first key enzyme of flavonoid pathway.

CHS, the best studied plants-specific type III ployketide synthase, catalyses the first committed step of the branch of the phenylpropanoid pathway which leads to the synthesis of flavonoids [25,26]. The product of the CHS reaction is a pivotal precursor for a vast array of secondary metabolites derived from malonyl-CoA and *p*-coumaroyl-CoA [27]. Enzymes of the CHS superfamily share high similarity in their amino acid sequence, structure and general catalytic principles and function as a homodimer (monomer size about 43 kDa) which contains the conserved Cys-His-Asn catalytic triad in the buried active sites [28]. More recently, the crystal structure and functional studies of alfalfa CHS2 have confirmed that the conserved cysteine and histidine residues play a significant role in the catalytic function of CHS, and site-directed mutants of these residues have been investigated to elucidate the reaction mechanism of CHS [29,30]. Furthermore, a previous study also showed that the reaction catalyzed by CHS can be seen as a three-step reaction which involves transfering the *p*-coumaroyl moiety to the active site residue Cys^164^, decarboxylation of malonyl-CoA to an acetyl-CoA carbanion and extending the polyketide intermediate [31].

Because of its essential role in flavonoid biosynthesis, *CHS* gene has been isolated and well characterized in hundreds of plant species, especially in eudicots [32]. Previous studies have also described the phenotypic and metabolic effects of loss of CHS protein activity in different model plant species. In 1936, the first *CHS* mutant identified in *Antirrhinum majus* was the chalk white nivea mutant which lacks flavonoids and possesses the white-flowered phenotype because of the deletion of the single *CHS* gene [33]. *White anther* (*wha*), a petunia *CHS* mutant, generated by using ethylmethanesulphonate is functionally male sterile with reduced corolla pigmentation. Biochemical and transgenic complementation studies by Napoli *et al*. demonstrated that *wha* mutation was in *CHS-A* gene which is expressed in petunia petal and pollen tissues [34]. In *Arabidopsis*, *tt4* mutants lacking CHS protein activities accumulate no brown condensed tannins in their seed coats and only the yellow colour of the underlying embryo is visible. Additionally, those mutants exhibited no anthocyanin pigments in the cotyledons or hypocotyls when grown in Murashige and Skoog (MS) media, while the seedlings of wild-type *Arabidopsis* had strong red pigmentation [35,36]. In consequence, these mutants are helpful to define the function of *CHS* gene and the roles of those compounds derived from flavonoid pathway.

*Freesia hybrida*, a monocotyledonous ornamental species, widely distributed in the world, and is becoming increasingly popular as a cut flower. The range of flower colours available in the freesias includes red, pink, yellow, white, blue, lavender, purple and bicolors. Thereby, the freesias are a good system for studying the secondary metabolism of flowers with different colors. Furthermore, the characterization of *CHS* gene from *F*. *hybrida* may help the studies of the duplication and evolution of the *CHS* gene family considering that there are only a few reports of *CHS* gene from Iridaceae.

In this paper, we first report the isolation of a *CHS* gene from *Freesia hybrida* and investigation of its spatial and temporal expression at different developmental stages and in different organs. Secondly, structure and enzyme activity is clarified using the purified recombinant protein from the *E*. *coli* system. Finally, functionality of this *CHS* gene is characterized via its ectopic expression in both *Arabidopsis tt4* mutants and petunia, and detailed alteration of flavonoids in flowers of transgenic petunia lines was also assayed. In summary, these results suggest the role of *FhCHS*1 in biosynthesis of flavonoid during floral coloration.

## Materials and Methods

### Plant materials

Wild-type, *Arabidopsis thaliana tt4* mutant, and T2 transgenic seedlings of *Arabidopsis* were germinated in the greenhouse (16 h of light/8 h of dark cycle, 22°C). Seven-day-old seedlings were collected and stored at -80°C. *Petunia hybrida* plants (Xinhui Horticulture Co., China) for transformation were grown aseptically on Murashige and Skoog medium supplemented with 3% sucrose, and transgenic flowers were harvested at the full-bloom stage. Tissues and flowers at different stages of development from *Freesia hybrida* were collected and stored at -80°C until required.

### Cloning of the full-length cDNA of *FhCHS1*

A cDNA library was constructed from petals of red *Freesia hybrida* using a pBluescript II XR Predigested Vector Kit (Invitrogen). Colonies were transferred onto Hybond-N+ nylon membranes, and then denatured and fixed. The polymerase chain reaction (PCR) fragment previously isolated from *Iris germanica* was used as a probe. The membranes were prehybridized for 1 h at 42°C before the probe was added and hybridized in the manufacturer’s hybridization buffer at 42°C overnight. Labeling of the probe was carried out according to the manufacturer’s instructions using an ECL direct nucleic acid labeling and detection system (Amersham). Positively hybridized plaques were isolated and sequenced, one of which was identified to be a CHS homologue with the 3′ end sequence of *FhCHS1*. Total RNA from petals of red *Freesia hybrida* was isolated using the RNAiso Plus (TaKaRa), and then was treated with RNase-free DNase I (TaKaRa), which was heat inactivated at 65°C for 10 min. SMARTer RACE cDNA Amplification Kit (Clontech-Takara, Catalogue no. 634923) was used to synthesise the first-strand cDNA from *Freesia hybrida* for 5′ RACE, according to the manufacturer’s protocol. After dilution, the first-strand reaction product was subjected to PCR for amplification. The gene-specific primers GSP (5′-CTCCGGTCGTACCGTTCTTCTCCT-3′) as reverse primer in combination with the universal primer A mix (UPM, provided in the kit) as the forward primer were used with the following cycling program: initial denaturation at 94°C for 5 min, followed by 30 cycles of 94°C for 30 s, 68°C for 30 s and 72°C for 2 min, and a final extension at 72°C for 5 min. The amplified PCR products were analyzed by electrophoresis on a 1% agarose gel, and then cloned into pGEM-T Easy Vector (Promega) to sequence. Once the 5′ sequences were determined, the full-length *FhCHS1* was amplified from the cDNA and genomic DNA with the following primer sequences, forward primer 5′-GCAAGAAAAATGGTGAATG-3′ and reverse primer 5′-TGGGAATGATATATAGGGAGTC-3′, in order to determine the accurate nucleotide sequence of cDNA and the presence of intron.

### CHS enzyme assays

The full-length open reading frame of *FhCHS1* cDNA was amplified by PCR using *Premix Taq* (TaKaRa) with the following primers: forward primer 5′-CGGGATCCAGAAAAATGGTGAATG-3′ (the *Bam*HI site is underlined) and reverse primer 5′-CGAGCTCTGGGAATGATATATAGGGAGTC-3′ (the *Sac*I site is underlined). The amplified fragment was then introduced into *Bam*HI/*Sac*I-digested pET-28a (+) expression vector, and the construct was sequenced to ensure that the gene was in the correct reading frame. After transformation into the *Escherichia coli* strain BL-21 (DE3), colonies were selected on LB medium containing 100 mg/L kanamycin (KAN) plates. Individual colonies were grown overnight in 4 mL of LB-KAN medium at 37°C, and 2 mL of the culture was used to inoculate 400 mL of LB-KAN fresh medium. Cells were grown to OD_600_ of 0.6 at 37°C and induced for 8 h with 0.55 mM isopropyl thio-*β*-D-galactoside at 25°C, followed by harvest by centrifugation at 4,000 g for 10 min and resuspended in 20 mL of phosphate-buffered saline. After sonication on ice, the supernatant and pellet were tested by SDS-PAGE for solubility of the fusion protein by Coomassie blue stain. Finally, the soluble proteins were purified using Ni Sepharose High Performance following the manufacturer’s instructions (GE Healthcare).

The purified recombinant FhCHS1 was used to examine enzyme activity with *p*-Coumaroyl-CoA (MicroCombiChem e.K., Wiesbaden, Germany) and Malonyl-CoA (Sigma) as substrates. The standard assay contained 80 μM starter CoA, 160 μM malonyl-CoA, 0.1 M potassium phosphate (pH 7.2), 30 μg of protein, and was incubated at 30°C for 1 h. Subsequently, reactions were extracted twice with 300 μl ethyl acetate and centrifuged at 10,000 g for 20 min. After drying under vacuum, the samples were dissolved in 100 μl of methanol. Meanwhile, BL-21 (DE3) cells transformed with an empty pET-28a (+) vector were used as a control and the resulting protein extract was assayed under the same conditions. For analysis of the product, the mixture was filtered with a 0.22 μm filter and analyzed by high-performance liquid chromatography (HPLC). The mobile phases were water (A) and methanol (B) at a flow rate of 0.6 ml min^-1^. Detection of the enzymatic products was carried out in the detection range of 289 nm by means of a linear gradient elution for 30% B for 3 min, 30–70% B for 22 min, 70–80% B for 2 min, 80–95% B for 3 min, and 95% B for 5 min.

### Crystallization and data collection

Recombinant FhCHS1 protein was affinity purified and concentrated to 7.4 mg/ml, then vapour diffusion crystallization drops was then set up at 2 μl drop size with 1:1 ratio of mixture between protein and crystallization mother liquor (0.2 M di-ammonium hydrogen citrate, 20% w/v PEG 3350). Bar shaped crystals were observed after up to 7 days of incubation at 4°C. Crystals were cryo-protected with cryo buffer (0.2 M di-ammonium hydrogen citrate, 20% w/v PEG 3350, 25% glycerol) before flash frozen at 105 K by liquid nitrogen.

Diffraction data was collected at energy of 14.2080 keV from beamline ID23–2 in European Synchrotron Radiation Facility, Grenoble, France. Typically, the obtained crystals diffracted to 2.6 Å of resolution, with a best case of 1.6 Å resolution. The CHS crystals belonged to the space group P212121 with unit cell dimensions of a = 233.76 Å, b = 63.45 Å, c = 108.66 Å, α = β = γ = 90°. Diffraction data was processed by xds [37]. Phase was obtained by carrying out Molecular Replacement in the program Phaser [38] by using PDB ID of 1BI5 as the search model. The generated model was further refined by the programme Phenix [39] with start Rwork = 0.2193, Rfree = 0.2957 and final Rwork = 0.2260, Rfree = 0.2645.

### Phylogenetic analysis

To construct the phylogenetic tree, the full-length amino acid sequences of CHS protein were aligned with the ClustalW2 using default parameters (http://www.ebi.ac.uk/Tools/msa/clustalw2/). The alignments were saved and executed by MEGA version 5.1 to generate a neighbor-joining tree with bootstrapping (2,000 replicates) analysis and handling gaps with pairwise deletion.

### Expression profile analysis

For real-time quantitative PCR, total RNA was extracted from different tissues and flowers of *Freesia hybrida*. This was done by grinding fresh tissue in liquid nitrogen to a fine powder and extracting in RNAiso Plus (TaKaRa), according to manufacturer’s protocol. Then total RNA extracted from the above samples was treated with RNase-free DNase I (TaKaRa) before cDNA synthesis.

A SYBR Green-based real-time PCR assay was carried out in a total volume of 10 μl of reaction mixture containing 5 μl of 2 × Master Mix (TOYOBO), 0.5 μM of each primer, and 1 μl cDNA. Cycling conditions were 95°C for 60 s, followed by 40 cycles of 95°C for 5 s and 60°C for 60 s. The primers FhCHS1F1 (5′-CACCAA CAGCGAGGATAA-3′) and FhCHS1R1 (5′-AGACATTCGGATTTTCCT-3′) were used for real-time PCR to amplify a 122-bp fragment. The *FhCHS1* expression data was normalized against the expression level of an internal control *18s rRNA* gene (forward primer 5′-TCCTGATACGGGGAGGTAGTGACA-3′, reverse primer 5′-ACTTGCCCTCCAATGGATCCTCG-3′) fragment with 111-bp long. The fidelity of the PCR products was confirmed by using agarose gel electrophoresis and sequencing. All analyses were repeated three times using biological replicates. Differences in cycle thresholds between target and *18s rRNA* genes corresponded to relative expression level of the target gene.

### Expression of *FhCHS*1 in *Arabidopsis* mutants

A pair of primers, 5′- CGGTCTAGAGCAAGAAAAATGGTGAAT-3′ (the *Xba*I site is underlined) and 5′-CGAGGATCCGTGCTACTTAAGCTTCA-3′ (the *Bam*HI site is underlined), was designed to amplify the whole coding regions of *FhCHS1* gene. The PCR products were digested with *Xba*I and *Bam*HI and then was subcloned into the vector pBI-121containing the constitutive cauliflower mosaic virus 35S promoter. *Arabidopsis thaliana tt4* mutants (*CHS*; At5g13930) were transformed using *Agrobacterium tumefaciens* strain GV3101 according to the floral dip techniques [40]. For transgenic plant selection, transformants were germinated on 1/2 MS medium containing 50 mg/L kanamycin. After selection, kanamycin-resistant plants with red cotyledons were transplanted to soil and grown in the greenhouse under long days. Total RNA extracted from T2 transgenic seedlings was used for reverse transcription PCR (RT-PCR) to confirm the expression of *FhCHS1* gene in the transformed mutant lines, and the *actin-1* gene was used as an internal control gene [41].

### Transformation of petunia by *Agrobacterium tumefaciens*

The vector used for *Arabidopsis* transformation was also used for petunia transformation by using an *Agrobacterium tumefaciens*-mediated leaf transformation protocol. Two-month-old petunia plants were used for transformation by following a previously described protocol, and selected with 50 mg/L of kanamycin [42]. After rooting, kanamycin-resistant plants were transferred to soil and grown in a greenhouse. To confirm the expression of *FhCHS1* gene in the transformed plants, total RNA was isolated from flowers of T0 transgenic plants and used in RT-PCR. The *18s rRNA* gene was used as a constitutive control with the following primer sequences: 5′-TACCACATCCAAGGAAGGCA-3′ and 5′-ACCCAAAGTCCAACTACGAG-3′.

### Anthocyanins and flavonols analysis

Anthocyanins and flavonols were extracted from 100 mg of finely ground tissues in 1 ml of extraction solvent mixture containing H_2_O:MeOH:HCl (75/24/1,v/v/v) at 4°C for 12 h in the dark. The extract was centrifuged at 10,000 rpm for 10 min at room temperature, and then supernatant was filtered with 0.22 μm filter and used for HPLC analyses. HPLC assay was performed on a Shimadzu HPLC system equipped with an ACCHROM XUnion 5 μm C18 column (250 × 4.6mm). The HPLC flow rate was set at 1 ml min^-1^ and the mobile phases are composed of A (5% formic acid in H_2_O) and B (methanol). The elution gradient was as follows: 0–10 min, 14–17% B; 10–35 min, 17–23% B; 35–60min, 23–47% B; 60–67 min, 47–14% B; 67–70 min, 14% B. HPLC separations were monitored at 520 and 360 nm. The HPLC-ESI-MS system adopted in our study consisted of an API2000 mass spectrometer (AB Sciex) and a SPD-20AV UV/VIS Dectector (Shimadzu, Kyoto, Japan). An Analyst (version 1.5, Applied Biosystems) was used for data acquisition and processing. The optimal settings of the mass spectrometer were as follows: positive mode, ion spray voltage 4,500 V, scan range of 100 to 1,000 m/z, declustering potential +80 V and entrance potential 10 V.

Quantification analysis was performed by comparison with commercial standards, including cyanindin-3-glucoside chloride and quercetin-3-glucoside (Sigma) [43]. Data were represented as mean values from at least three independent experiments with three replicates each. Statistical significances of the differences were determined using Student’s *t*-test. Differences between wild-type and transgenic lines were considered significant when P< 0.01.

## Results

### Sequences and structure analysis of FhCHS1

**Fig 1:**
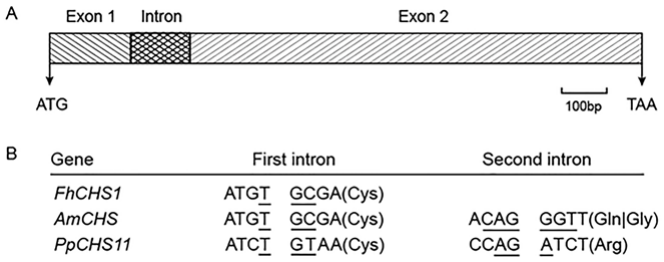
Schematic diagram of the generalized structure of *FhCHS1* gene. A, Exons-intron architecture of *FhCHS1*. exons, intron, initiation codon (ATG) and terminator codon (TAA) are labeled. B, Nucleotide sequences surrounding splice sites in *FhCHS1*, *AmCHS* (*Antirrhinum majus CHS*, X03710) and *PpCHS11* (*Physcomitrella patens CHS*, ABU87504). Amino acid residues and codons (underlined) split by introns are shown.

**Fig 2:**
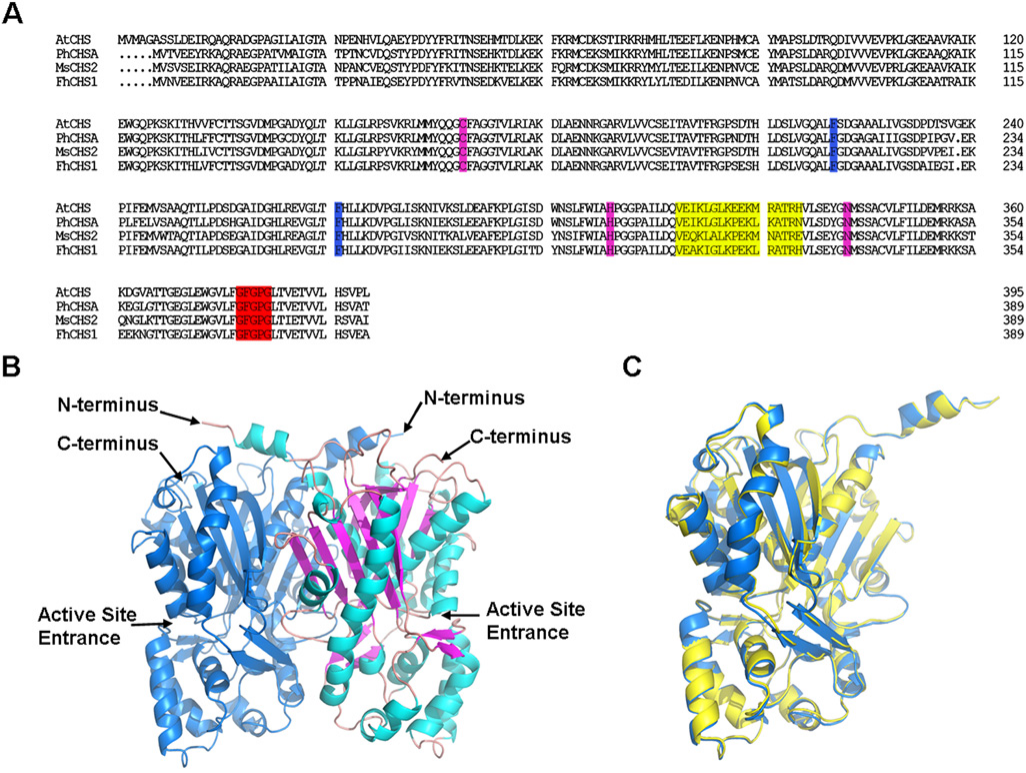
Sequence and crystal structural analysis of FhCHS1. A, Comparison of amino acid sequences between FhCHS1, AtCHS (At5913930), PhCHSA (X14591) and MsCHS2 (L02902). Functionally important conserved residues are highlighted with a colored background: pink, the three conserved catalytic residues in all CHS; blue, two important residues determining the substrate specificity of CHS; yellow, the malonyl-CoA binding motif; red, highly conserved CHS signature sequence. B, Functional unit of CHS. The homo-dimer with one of the monomer highlighting secondary structure components: cyan for α-helix, magenta for β-strand, pink for loop. C, Structure comparison of the current CHS of freesia (blue, PDB ID 4WUM) and previous structure model CHS2 of alfalfa (yellow, PDB ID 1BI5), indicating strong structural conservation of the CHS protein.

**Fig 3:**
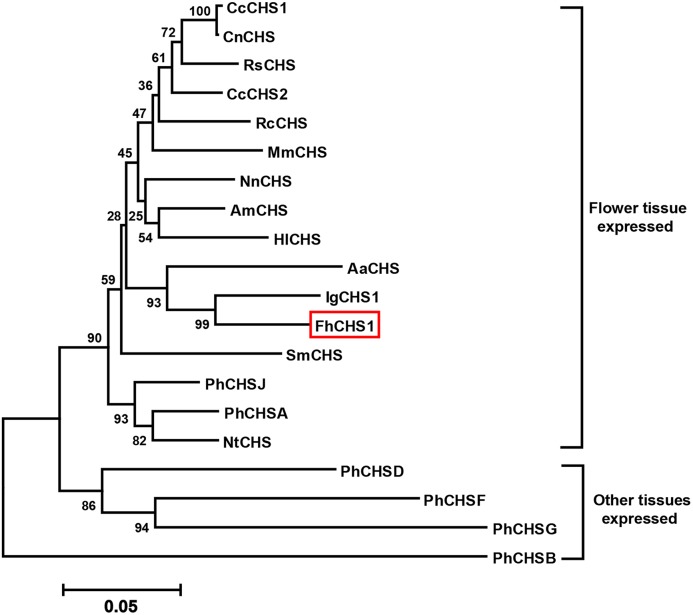
Phylogenetic tree based on the amino acid sequences of plant CHSs. The tree was constructed using the MEGA 5.1 and neighbor-joining method with 2000 bootstrap replicates. *Freesia hybrida* CHS is boxed. GenBank accession numbers used are: *Nelumbo nucifera* (*Nn*CHS, FJ999628.1), *Abelmoschus manihot* (*Am*CHS, EU573212.1), *Nicotiana tabacum* (*Nt*CHS, AF311783.1), *petunia hybrida* (*Ph*CHSA, X14591; *Ph*CHSB, X14592; *Ph*CHSD, X14593; *Ph*CHSF, X14594; *Ph*CHSG, X14595; *Ph*CHSJ, X14597), *Saussurea medusa* (*Sm*CHS, DQ350888.1), *Humulus lupulus* (*Hl*CHS, CAK19317.1), *Anthurium andraeanum* (*Aa*CHS, DQ421809.1), *Freesia hybrida* (*Fh*CHS1, JF732897.1), *Iris germanica* (*Ig*CHS, AB219147.1), *Camellia chekiangoleosa* (*Cc*CHS1, JN944573.1; *Cc*CHS2, JN944574.1), *Rosa chinensis* (*Rc*CHS, HQ423171.1), *Melastoma malabathricum* (*Mm*CHS, KF234569.1), *Rhododendron simsii* (*Rs*CHS, AJ413277.1), *Camellia nitidissima* (*Cn*CHS, HQ269804.1).

Through heterologous screening of a cDNA library and PCR amplication, cDNA (deposited in GenBank, accession no. JF732898) and genomic sequence of *FhCHS1* gene were obtained. The gene has one intron with an open reading frame of 1,170 bp encoding 389 amino acid residues with a calculated molecular mass of 42.58 kDa and an isoelectric point of 5.74 (Fig. 1A). The intron of *FhCHS1* begins with the nucleotides ‘GT’ and ends with the nucleotides ‘AG’ (Fig. 1B), and the intron length was 129 nt (S1 Fig.), following the GT-AG rule. Multiple sequence alignments revealed that FhCHS1 shared 83.55% to 85.86% identity with AtCHS, PhCHS and MsCHS2 (Fig. 2A). Consistent with previous results with other CHS proteins, the FhCHS1 protein also contained the three important catalytic residues of Cys^164^, His^303^, and Asn^336^ (marked with pink background) and two highly conserved Phe residues, Phe^215^ and Phe^265^ (marked with blue background), which were identified to be important for the formation of the active site as well as for the substrate specificity of the CHS. Additionally, the malonyl-CoA binding motif (marked with yellow background) and a highly conserved CHS signature sequence (marked with red background) were also found in the FhCHS1 sequence (Fig. 2A). As shown in Fig. 2B&C, the crystallographic symmetry of FhCHS1 is a dimer of dimer, with the homodimer as the biological functional unit. The individual monomer consists of roughly two domains with the active site located at the clef between the two domains and the dimerization interface, therefore sharing the accessible substrate binding area with the dimer partner. On the other hand, FhCHS1 belonged to the cluster of CHSs expressed in flower tissue and was most closely related to CHS from *Iris germanica* (Fig. 3) in the phylogenetic analysis.

### Enzymatic activity of freesia CHS proteins

**Fig 4:**
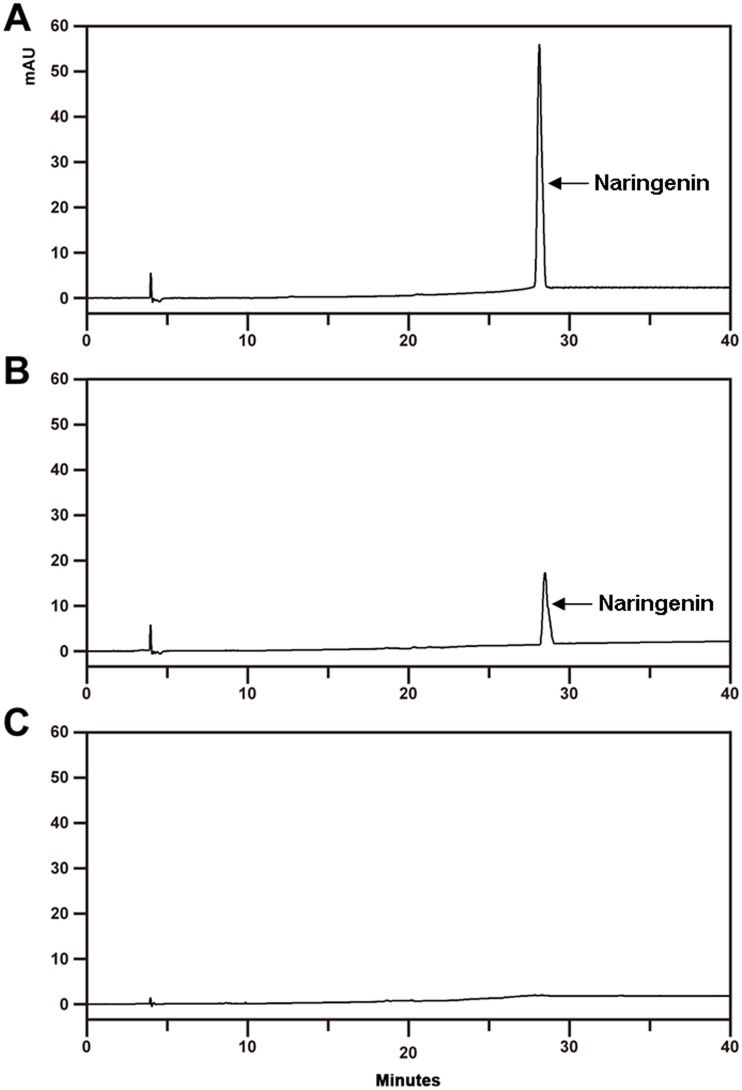
Enzyme activity of freesia (*Freesia hybrida*) chalcone synthase (FhCHS1). A, The sample of naringenin standard. B, The reaction products catalyzed by FhCHS1. C, Control.

In order to examine the catalytic properties of FhCHS1, it was heterologously expressed in *E*. *coli* with an additional his-tag at the N terminus. Enzyme activity of FhCHS1 was tested with *p*-coumaroyl-CoA, malonyl-CoA and purified protein, and the reaction products were analyzed by HPLC in comparison to authentic standards. As well known, naringenin chalcone is converted non-enzymatically to naringenin in the course of the reactions performed *in vitro*, and therefore naringenin is the main product in the analysis of HPLC. Compared with control, the reaction product generated a new peak at a retention time (28.5 min) that matched the authentic naringenin (Fig. 4). This indicates that FhCHS1 has a typical CHS enzymatic function, catalyzing the conversion of *p*-coumaroyl-CoA and malonyl-CoA substrates to naringenin chalcone.

### Expression patterns of *FhCHS*1 in developing flowers and other tissues

**Fig 5:**
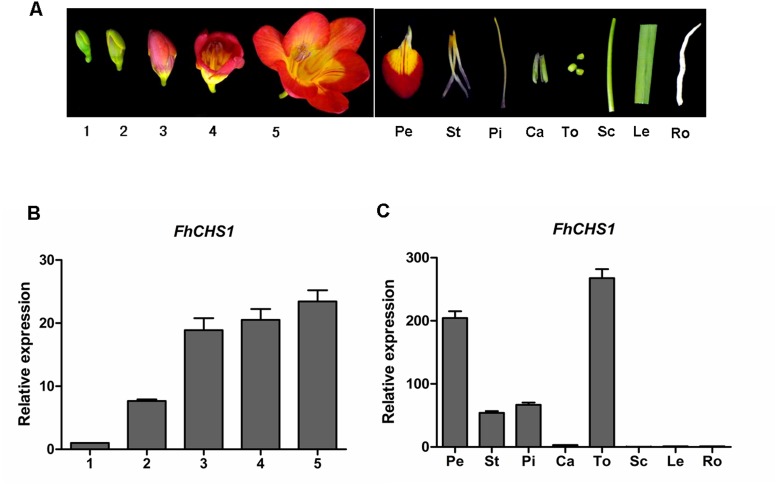
*FhCHS1* gene expression profiles in *Freesia hybrida*. A, The phenotypes of different samples. 1–5, represent the flowers of different developmental stages; Pe, petals; St, stamens; Pi, pistils; Ca, calyxes; To, toruses; Sc, scapes; Le, leaves; Ro, roots. B, Expression profile of *FhCHS*1 in flowers at different developmental stages. C, Expression levels of *FhCHS*1 in different tissues. Data represent means ± SD of three biological replicates.

Expression pattern of *FhCHS1* was examined using quantitive real-time qPCR. Total RNA was extracted from different freesia tissues including flowers at five developmental stages (stage 1, <10 mm long with unpigmented buds; stage 2, 10–20 mm long with slightly pigmented buds; stage 3, 20–30 mm long with pigmented buds; stage 4, fully pigmented flowers before complete opening; stage 5, fully opened flowers), and different tissues including petals, stamens, pistils, calyxes, toruses, scapes, leaves and roots (Fig. 5A). Overall, transcripts of *FhCHS1* were detected in all examined samples at different expression levels. The expression level of *FhCHS1* gradually increased during flower development and peaked at stage 5 (Fig. 5B). And this expression profile of *FhCHS1* during developmental stages corresponded to the accumulation of total anthocyanin during flower development. Furthermore, the transcript level of *FhCHS1* was also found significantly higher in flower organs than those in vegetative organs as shown in Fig. 5C. The experiment data showed that *FhCHS1* reached its highest expression level in torus and then decreased to a very low level in leaves and roots. Taken together, these findings indicated that *FhCHS1* was involved in the development of pigmentation in flower and biosynthesis of flavonoid in other tissues.

### Complementation of CHS function in *Arabidopsis tt4* mutants

**Fig 6:**
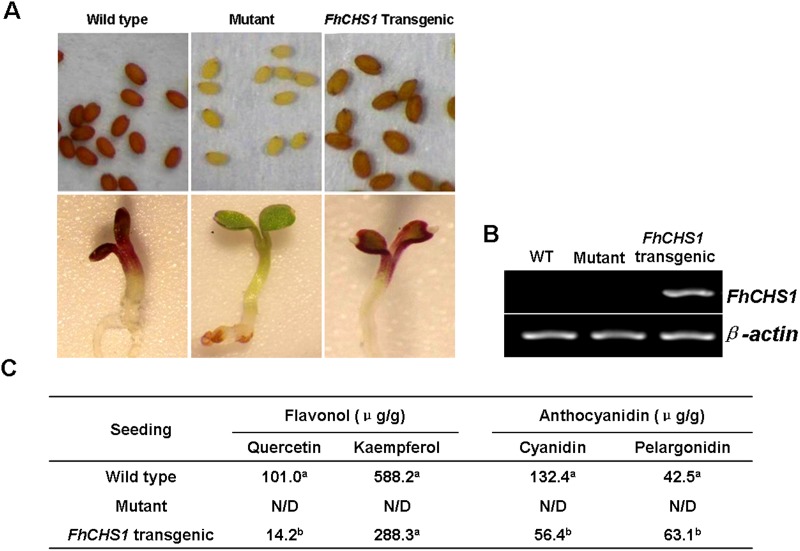
Complementation of the pigmentation of *Arabidopsis tt4* mutant seedlings with *FhCHS1* gene. A, Phenotypes of wild-type, *tt4* mutant, and transgenic *Arabidopsis* seeds and seedlings. B, Expressional analysis of the *FhCHS1* gene by reverse transcription polymerase chain reaction in the wild-type, *tt4* mutant and transgenic lines. C, Contents of anthocyanidins and flavonols in *Arabidopsis* seedlings. Data correspond to means of three biological replicates. Means with different letters within the same column are significantly different at the 0.01 level of probability. N/D, not detected.

In order to examine the *FhCHS1* function in flavonoid biosynthesis, the cDNA was transferred into the *Arabidopsis tt4* mutants under the control of the cauliflower mosaic virus 35S promoter. The *Arabidopsis tt4* mutants cannot synthesize tannins in their seed coats as well as anthocyanins in cotyledons and hypocotyls (Fig. 6A). After transformation, several T1 kanamycin-resistant plants showed pigmentation at the rosette base similar to the wild type *Arabidopsis*, whereas *Arabidopsis tt4* mutant lacked the visible pigments (S2 Fig.). Additionally, seeds collected from these T1 plants exhibited pigmentation characteristic of the wild-type *Arabidopsis*. When the T2 seeds were grown on 1/2 MS medium containing 3% sucrose, germinating seedlings regained the red cotyledons compared with the green cotyledons in *Arabidopsis tt4* mutants (Fig. 6A), and this could be caused by the expression of *FhCHS1* (Fig. 6B). Furthermore, 100 mg of T2 seedlings were extracted with extraction solvent mixture and analyzed by HPLC to determine the amounts of individual anthocyanins and flavonols (Table A in S1 File). The results showed that *tt4* mutant had reduced peak area for all the peaks of anthocyanin (monitored at 520) and some peaks of flavonol (monitored at 360) relative to the wild type control, as anticipated, T2 seedlings carrying *FhCHS1* cDNA could restore these peaks (S3 Fig.). As for specific components, T2 seedlings had higher levels of pelargonidin but lower levels of cyanidin, kaempferol and quercetin than wild-type *Arabidopsis* (Fig. 6C). Overall, these results clearly demonstrated that *CHS1* gene from *Freesia hybrida* was fully functional in flavonoid biosynthesis in *Arabidopsis*.

### Overexpression of *FhCHS1* gene in petunia

**Fig 7:**
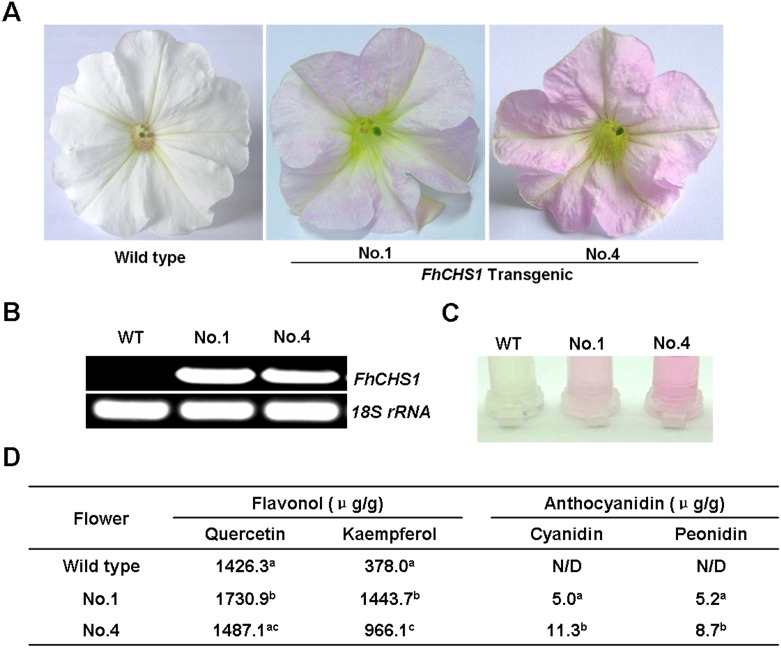
Functional characterization of *FhCHS1* gene following its overexpression in transgenic petunia lines. A, Differences in color between wild-type and transgenic petunia flowers. B, Expressional analysis of the *FhCHS1* gene by reverse transcription polymerase chain reaction in the wild-type and transgenic lines. C, Extracted solutions from flowers of wild-type and transgenic lines. D, Contents of anthocyanidins and flavonols in the wild-type and transgenic petunia flowers. Data correspond to means of three biological replicates. Means with different letters within the same column are significantly different at the 0.01 level of probability. N/D, not detected.

The coding region of *FhCHS1* was also transferred into petunia with white flowers, and a total of five transgenic lines were obtained. Among them, two lines (clone No. 1 and 4) showed significant changes of flower color (Fig. 7A). Thus, these two lines were subjected to further analyses, and the results showed that ectopic expression of *FhCHS1* (Fig. 7B) in petunia could alter the flower color from white to pink. Furthermore, the anthocyanin and flavonol composition in flowers of transgenic lines was also analyzed in detail (Table B in S1 File and S4 Fig.). The transgenic flowers showed markedly increased accumulation of cyanidin and peonidin derivatives reflecting by the color intensities of the extracted solutions (Fig. 7C). In contrast, the level of anthocyanidin pigments in wild type flowers was undetectable. On the other hand, flowers of those transgenic lines also exhibited notably higher levels of kaempferol derivatives than those of wild type petunia, while the content of quercetin derivatives was scarcely increased (Fig. 7D). Taken together, these results demonstrated that ectopic expression of *FhCHS1* gene promoted the biosynthesis of anthocyanins and flavonols in petunia flowers.

## Discussion

In addition to several other biological functions, flavonoids are important secondary metabolites that contribute to the appearance and quality of many flowers and fruits. The biosynthetic pathway of flavonoid has been extensively investigated in different model and economically important plant species, such as potato [44], grape [45] and apple [46]. In many plant species, core enzymes in the flavonoid pathway, such as CHS and DFR, are encoded by a gene family with up to 10 members [47], and it is deduced that each gene family was derived as a result of gene duplication events and subsequent functional divergence drove by positive selection [48]. Deng *et al*. found that an ancient duplication occurred in a common ancestor of monocots and eudicots, and gene families of the species tested in phylogenetic analysis are subject to various evolutionary fates [49]. However, in their study, the monocots they selected are exclusively from Poales only. In order to further trace the origination and evolution of the *CHS* gene family in angiosperm, it is crucial to isolate and characterize related gene families from other monocotyledonous species. The Iridaceae, one of the largest families of the monocots, is an ideal target, because the family comprises plants with large, brightly colored flowers, and is one of the most important plant families in horticulture [50]. Indeed, we have found additional *CHS* genes in *Freesia hybrida* through transcriptome analysis (unpublished data, available when requested), and *FhCHS1* is the one that is expressed dominantly in the red pigmented flowers. Here, we extensively studied the *FhCHS1*, and found that it is significantly responsible for biosynthesis of flavonoid in *Freesia hybrida*.

Firstly, an *FhCHS1* cDNA which had one intron was successfully isolated (Fig. 1A). Numerous studies have reported that most plant *CHS* superfamily genes contain a single intron that splits the conserved Cys in the consensus sequence of (K/Q)R(M/I)C(D/E)KS, while the second intron is not shared by all [13,51]. Furthermore, *CHS* genes which contain two introns also have the first intron at the conserved Cys and position of the second intron is situated within the codons for conserved amino acid residues such as Gly in *AmCHS* [52] and Arg in *PpCHS11* [13]. In agreement with the previous studies, the *CHS* gene from *Freesia hybrida* contained one intron and two exons, and the intron was inserted in the first cysteine codon as in almost all the *CHS* genes reported (Fig. 1B). Protein sequence alignment and structure analysis indicated the strong conservation of the sequence and structure of CHS protein, and these results provided some evidence for its enzymatic function [28].

The catalytic activity of the CHS enzyme has been detected in cell suspension cultures of parsley (*Petroselinum hortense*) by Kreuzaler *et al*. in 1972 [53]. In our study, molecular weight of the purified enzyme was about 43 KDa which is similar to CHS from *Hypericum androsaemum* and *Polygonum cuspidatum* [51,54]. Then enzyme assay using *p*-coumaroyl-CoA and malonyl-CoA as substrates demonstrated that FhCHS1 performed a typical CHS function (Fig. 4). Recently, Jiang *et al*. reported that PpCHS was able to convert *p*-coumaroyl-CoA and malonyl-CoA to naringenin chalcone as a major product together with two derailment products (*p*-coumaroyltriacetic acid lactone and bisnoryangonin) [55]. However, the two derailment products were not detected in our study, likely due to that the enzyme reaction mixture was not acidified prior to extraction. Next, further experiments are needed to examine whether FhCHS1 can convert *p*-coumaroyl-CoA and malonyl-CoA to other products such as *p*-coumaroyltriacetic and bisnoryangonin. In addition, it has been reported that CHS has broad substrate preference toward aromatic and aliphatic CoA esters [56]. For example, CHS from *Physcomitrella patens* has the ability to convert cinnamoyl-CoA and dihydro-*p*-coumaroyl-CoA to the corresponding chalcones. Among the CoA esters investigated, however, the most preferred substrate for the cyclization reaction of PpCHS is still *p*-coumaroyl-CoA [55]. Similarly, substrate specificity analysis of CHS from *Scutellaria baicalensis* also revealed that it could accept both aromatic and aliphatic CoA esters such as benzoyl-CoA, phenylacetyl-CoA, isovaleryl-CoA and isobutyryl-CoA to produce various reaction products including the unnatural aromatic polyketide [57]. In light of foregoing findings, further studies are needed to investigate the catalytic efficiency of FhCHS1 toward different substrates so as to confirm its *in vivo* substrate. Moreover, enzymatic analysis is also a vital tool to investigate the functional divergence of a gene family after duplication [58].

Real-time PCR results indicated that transcript level of *FhCHS1* was related to the stage of floral development with gradually increased expression pattern during flower blossoming, and this pattern showed significant correlation with the flower anthocyanin accumulation (Fig. 5B). This result is in line with the previous studies of wheat, in which the *CHS* expression in purple pericarp of caryopsis exhibited a growing trend during maturation [59]. Expression analysis in different developmental stages illustrates the fact that FhCHS1 is a key enzyme in the anthocyanin biosynthetic pathway as described by Moore *et al*. [60]. Subsequent analysis demonstrated that relatively low *FhCHS1* expression was observed in non-pigmented organs with the exception of torus (Fig. 5C). This suggests that *FhCHS1* is not flower specific and can be involved in the biosynthesis of other flavonoids.

Transfer of *FhCHS1* into the *Arabidopsis tt4* mutant successfully restored the pigmentation of their seed coats and purple coloration in the cotyledons and hypocotyls, confirming the *in vivo* function of FhCHS1 as a CHS (Fig. 6A). In the past years, complementation of *Arabidopsis* flavonoid mutants has revealed the functional conservation of enzymes involved in biosynthesis of flavonoid such as dihydroflavonol 4-reductase from sweet potato [61] and flavonoid 3-*O*-glucosyltransferase from black soybean [62]. Recently, the maize *C2* gene encoding CHS was expressed in *tt4* plants under the control of cauliflower mosaic virus 35S promoter, and transgenic seedlings showed similar patterns of flavonoid accumulation as those of wild-type *Arabidopsis* [63]. In accordance with above result, both the pigmentation phenotype and accumulation of anthocyanins and flavonols in *FhCHS1* transgenic seedlings were similar to the wild-type *Arabidopsis* (Fig. 6). Taken as a whole, these findings provide evidence that CHS proteins in the flavonoid biosynthesis are functionally exchangeable among different plant species.

Comparing to wild-type petunia, transgenic flowers carrying *FhCHS1* abundantly accumulated flavonol derivatives together with colored anthocyanins. As shown in Fig. 7D, flowers of transgenic petunia only accumulated cyanidin-based pigments, and the proposed pathway in transgenic petunia flowers was presented in S5 Fig.. The content of kaempferol derivatives in flowers expressing *FhCHS1* was enhanced approximately two to threefold. Meanwhile, a slight increase of quercetin derivatives was also observed. Dihydroflavonols, the common precursors for anthocyanins and flavonols, are substrates for both DFR and FLS [22]. As well known, DFR from petunia cannot accept the dihydrokaempferol as a substrate [64]. Therefore, the formation of pelargonidin-based pigments in transgenic petunia flowers was blocked, and the increased conversion of dihydrokaempferol to kaempferol derivatives by FLS might have occurred. By contrast, when the biosynthesis of cyanidin-based pigments was activated, accordingly, the increase in the contents of quercetin derivatives would be relatively lower than those of kaempferol derivatives. In *Arabidopsis*, it has been reported that disruption of DFR could enhance accumulation of flavonol [65], while *fls1* mutants showed increased anthocyanin levels [66,67]. Our results are consistent with these findings and reveal that substrate competition between FLS and DFR controls metabolic flux in the flavonoid pathway. In addition, transgenic flowers showed color alteration from white to pink and accumulated higher levels of cyanidin-based pigments than those of wild-type petunia. The results suggest that manipulation of the *FhCHS1* gene will contribute to modification of coloration in other plants.

In conclusion, we have successfully isolated *FhCHS1*, a *CHS* gene from *Freesia hybrida*, and examined its biological function in flavonoid biosynthesis. In addition, overexpression of *FhCHS*1 in petunia has increased the total amount of flavonoids in flowers. This clearly demonstrates that *FhCHS1* plays a crucial role in flavonoid biosynthesis and can be used to alter the composition of flavonoids of flowers to improve their aesthetic values. Furthermore, the characterized gene will probably contribute to the investigation of the origination and evolution of *CHS* gene families in angiosperm.

## Supporting Information

S1 Fig The full-length sequence of *FhCHS1* gene. The start condon (ATG) and the stop condon (TAA) are highlighted with red background, and the intron is underline.
(TIF)

S2 Fig The phenotypes of T1 transgenic *Arabidopsis* plants. (TIF)

S3 Fig HPLC analyses of anthocyanins and flavonols in transgenic *Arabidopsis* seedlings. (A-F) HPLC chromatograms of the samples from seedlings of WT, mutant and transgenic lines. (A, D) WT, (B, E) Mutant, (C, F) Transgenic lines. (A-C) Absorbance at 520 nm for analysis of anthocyanins. (D-F) Absorbance at 360 nm for analysis of flavonols.
(TIF)

S4 Fig HPLC analyses of anthocyanins and flavonols in transgenic petunia flowers. (A-F) HPLC chromatograms of the samples from flowers of WT and transgenic lines. (A, D) WT, (B, E) NO.1, (C, F) NO.4. (A-C) Absorbance at 520 nm for analysis of anthocyanins. (D-F) Absorbance at 360 nm for analysis of flavonols.
(TIF)

S5 Fig Proposed flavonoid pathway in transgenic petunia flowers. Hoary areas indicate biosynthesis of pelargonidin-based pigments is blocked.
(TIF)

S1 File Table A. HPLC-DAD and HPLC-ESI-MS analysis of flavonoid in acidic MeOH-H_2_O extracts of the wild-type *Arabidopsis* and *FhCHS1* over-expressing lines; Table B. HPLC-DAD and HPLC-ESI-MS analysis of flavonoid in acidic MeOH-H_2_O extracts of the wild-type petunia and *FhCHS1* over-expressing lines. (DOC)
