# Supplementary figures and images for: Molecular and Biochemical Analysis of Chalcone Synthase from Freesia hybrid in Flavonoid Biosynthetic Pathway
Source: PLoS One. 2015 Mar 5;10(3):e0119054. doi: 10.1371/journal.pone.0119054 (PMC4351062; doi:10.1371/journal.pone.0119054)

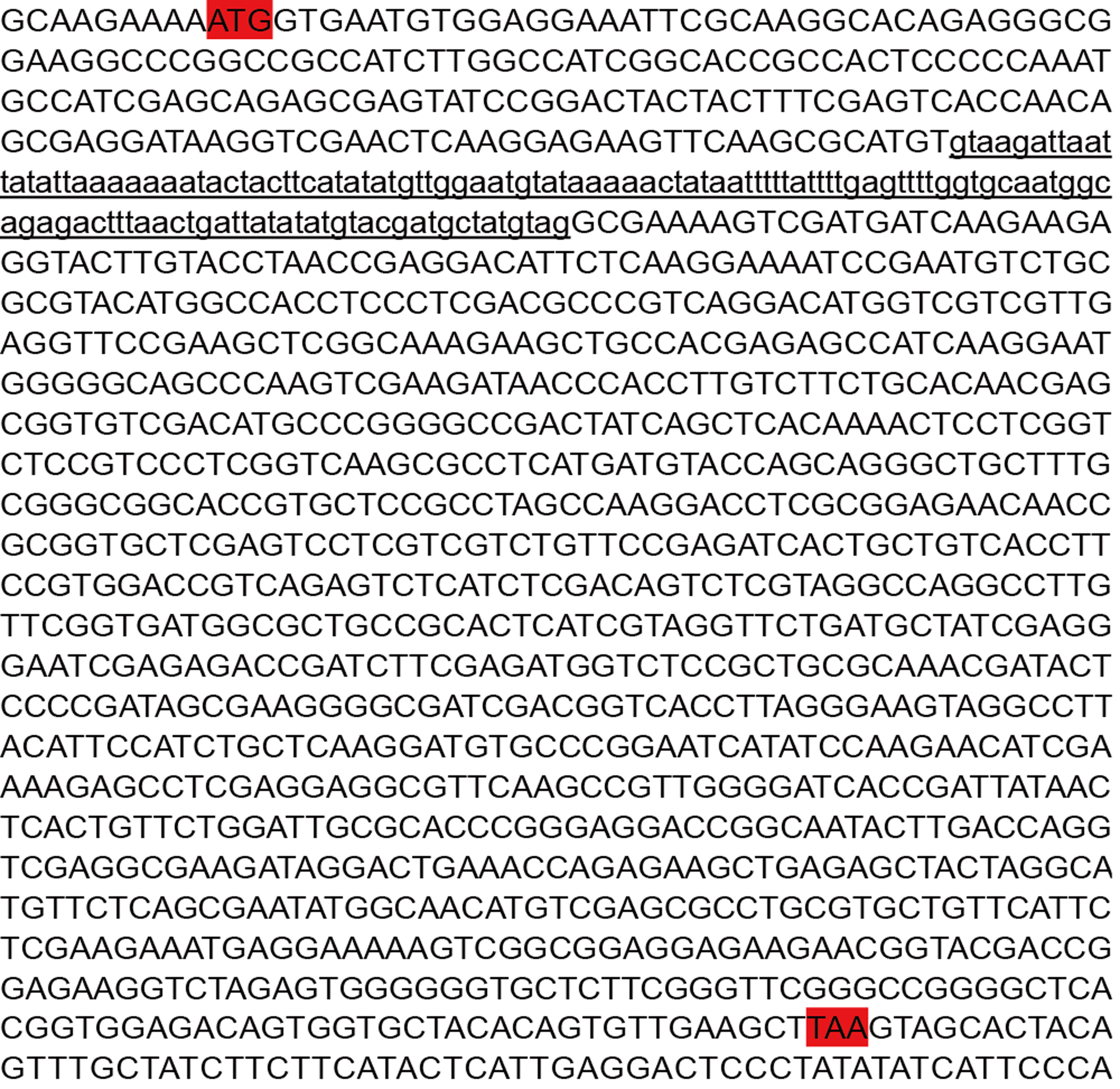

Supplement: S1 Fig — The start condon (ATG) and the stop condon (TAA) are highlighted with red background, and the intron is underline. (TIF) [file pone.0119054.s001.tif]

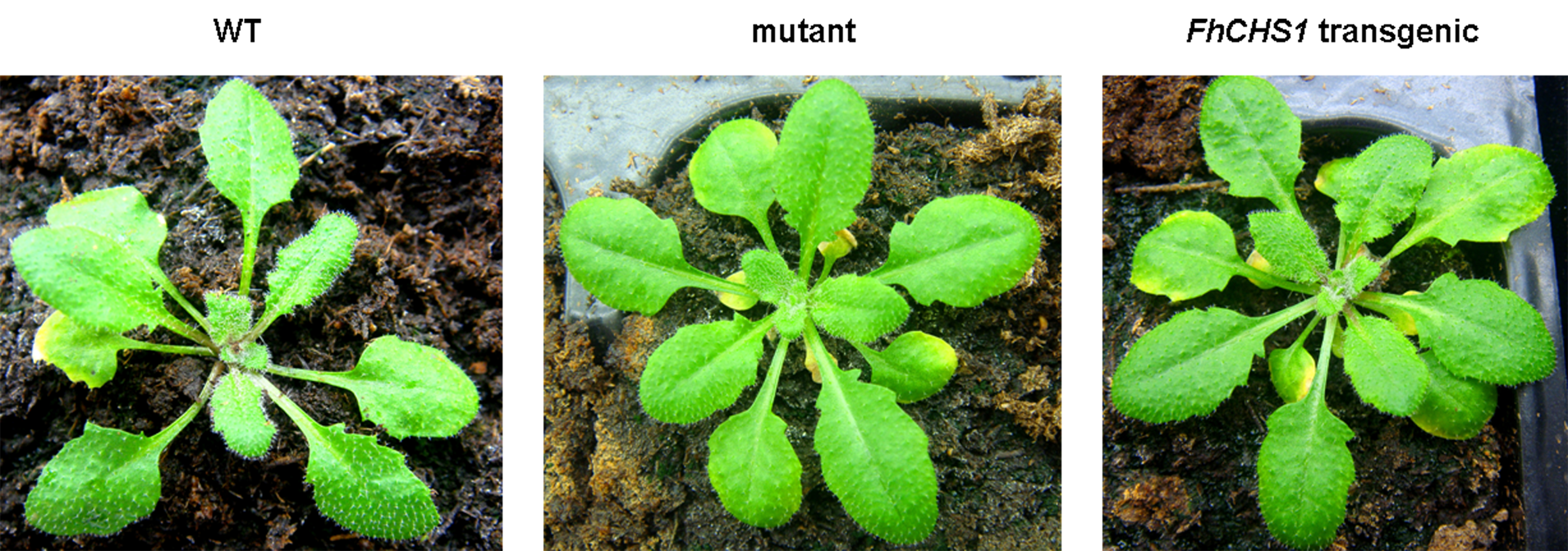

Supplement: S2 Fig — (TIF) [file pone.0119054.s002.tif]

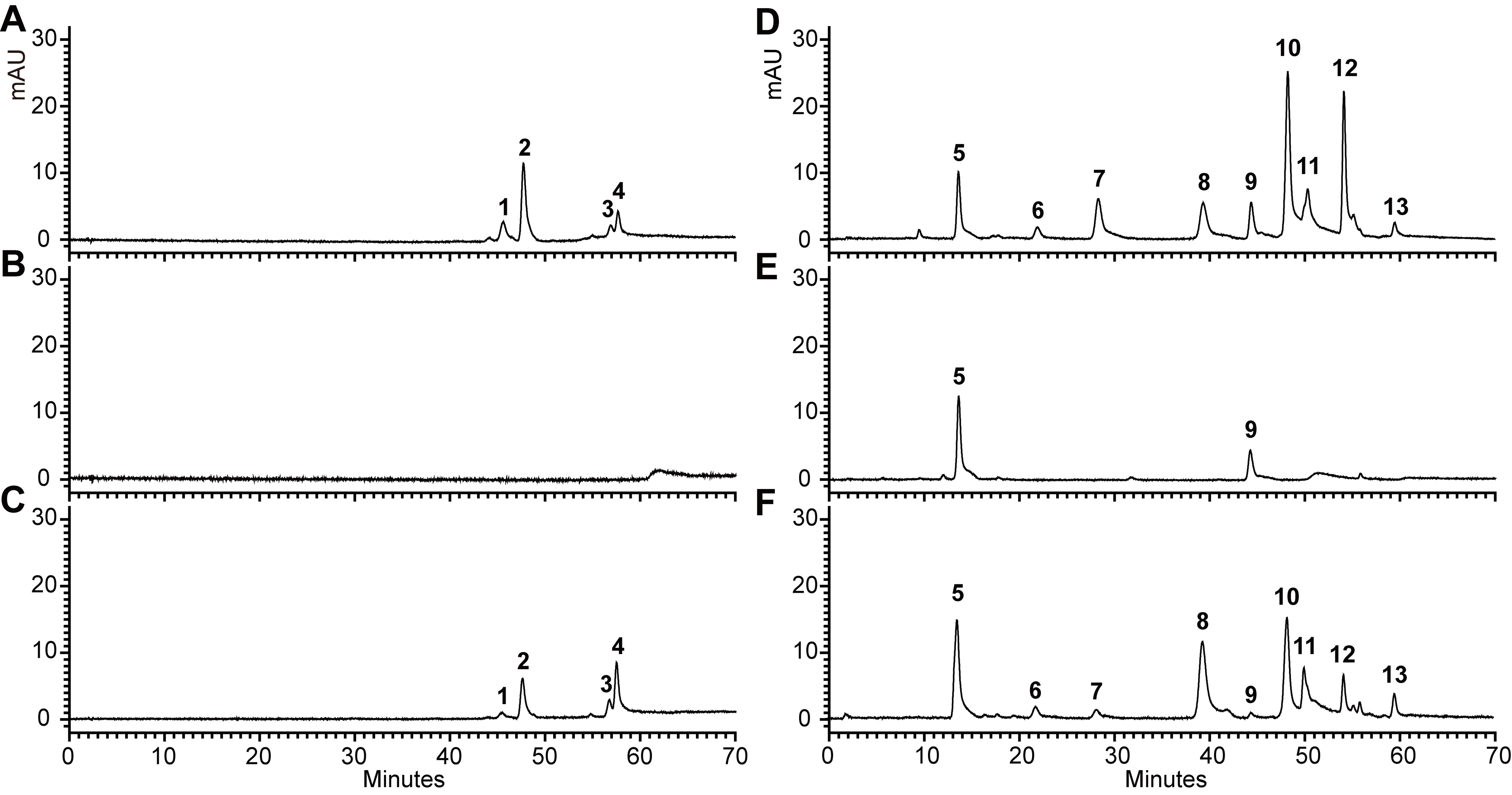

Supplement: S3 Fig — (A-F) HPLC chromatograms of the samples from seedlings of WT, mutant and transgenic lines. (A, D) WT, (B, E) Mutant, (C, F) Transgenic lines. (A-C) Absorbance at 520 nm for analysis of anthocyanins. (D-F) Absorbance at 360 nm for analysis of flavonols. (TIF) [file pone.0119054.s003.tif]

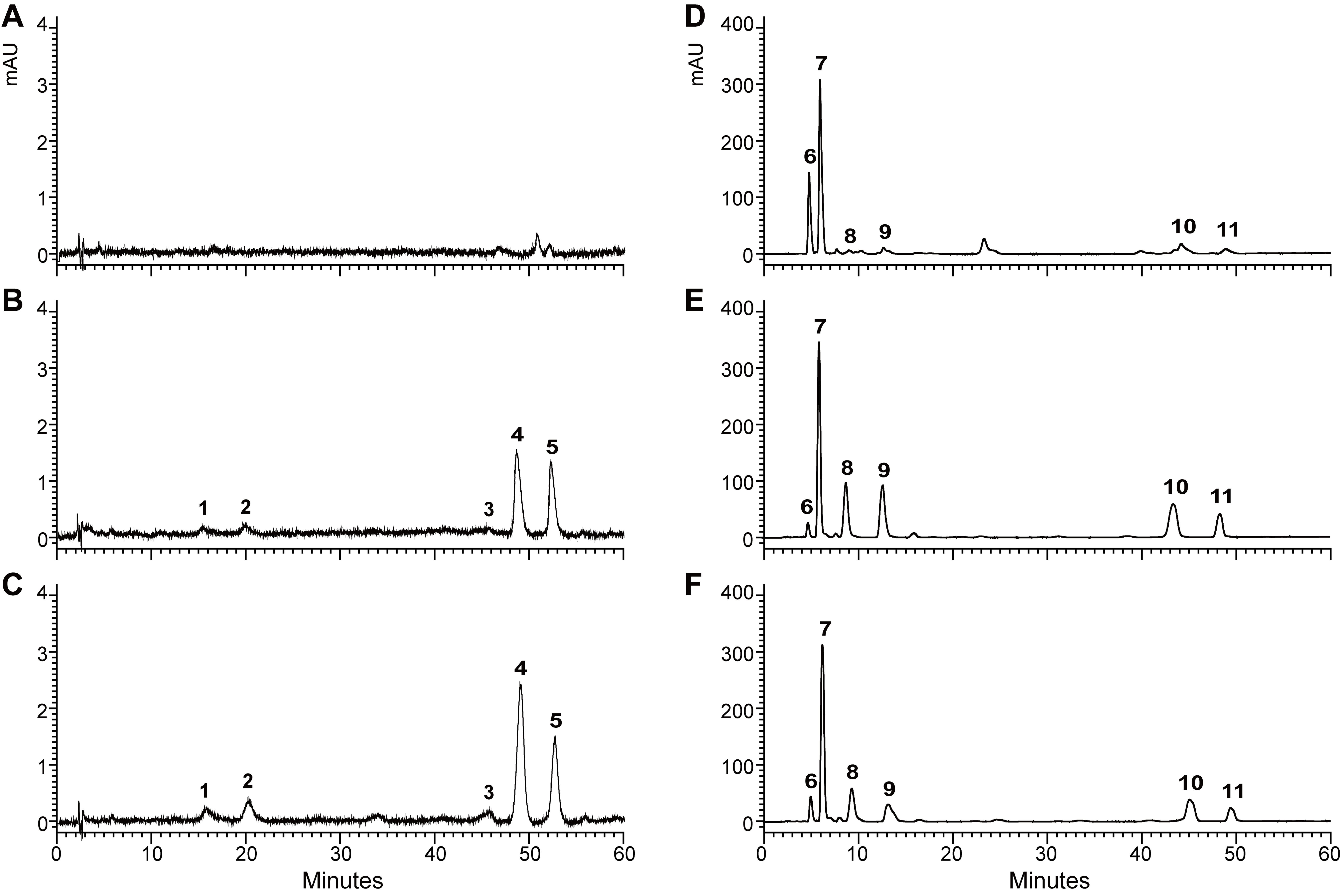

Supplement: S4 Fig — (A-F) HPLC chromatograms of the samples from flowers of WT and transgenic lines. (A, D) WT, (B, E) NO.1, (C, F) NO.4. (A-C) Absorbance at 520 nm for analysis of anthocyanins. (D-F) Absorbance at 360 nm for analysis of flavonols. (TIF) [file pone.0119054.s004.tif]

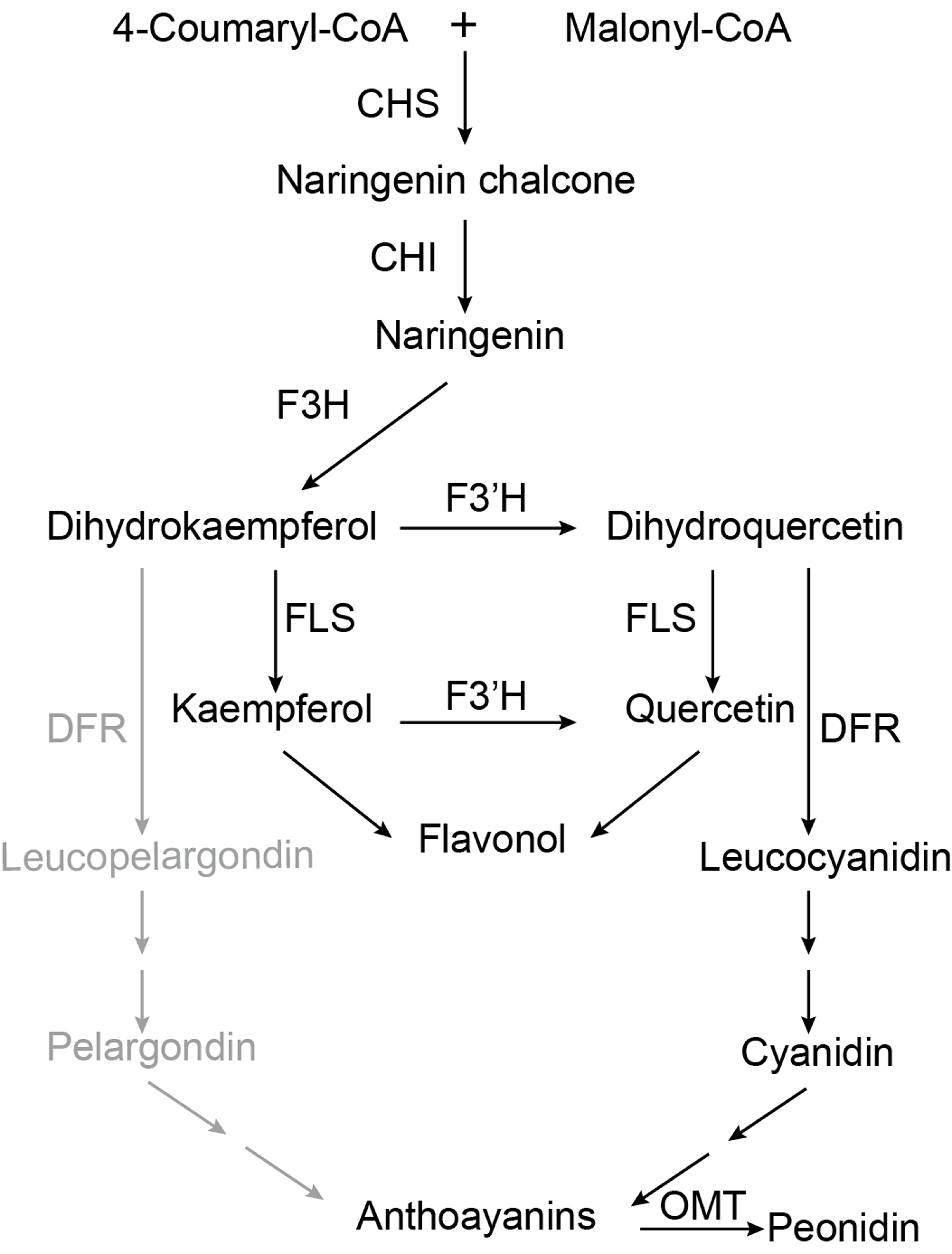

Supplement: S5 Fig — Hoary areas indicate biosynthesis of pelargonidin-based pigments is blocked. (TIF) [file pone.0119054.s005.tif]
