# Supplementary material for: Molecular and Biochemical Analysis of Chalcone Synthase from Freesia hybrid in Flavonoid Biosynthetic Pathway
Source: PLoS One. 2015 Mar 5;10(3):e0119054. doi: 10.1371/journal.pone.0119054 (PMC4351062; doi:10.1371/journal.pone.0119054)
Supplement: S1 File — (DOC) [file pone.0119054.s006.doc]

**Table A. HPLC-DAD and HPLC-ESI-MS analysis of flavonoid in acidic MeOH-H_2_O extracts of the wild-type *Arabidopsis* and *FhCHS1* over-expressing lines**

| **Peak number** | **Identifacation/tentative**  **identification** | **Retention time (min)** | **λmax (nm)** | **ESI-MS (m/z)** | **References** |
| --- | --- | --- | --- | --- | --- |
| **1** | **Cyanidin 3-*O*-[2*''*-*O*-(xylosyl) 6*''*-*O*-(*p*-*O*-(glucosyl) *p*-coumaroyl) glucoside] 5-*O*-[6*''''*-*O*-(malonyl) glucoside]** | **45.565** | **266**  **526** | **287.1[Cy+H]^+^**  **1137.4[M+H]^+^** | **Takayuki Tohge, Yasutaka Nishiyama et al., 2005** |
| **2** | **Cyanidin 3-*O*-[2*''*-*O*-(6*'''*-*O*-(sinapoyl) xylosyl) 6*''*-*O*-(p-*O*-(glucosyl)-*p*-coumaroyl) glucoside] 5-*O*-(6*''''*-*O*-malonyl) glucoside** | **47.720** | **294**  **534** | **287.0[Cy+H]^+^**  **1343.2[M+H]^+^** | **Stephen J. Bloora, Sharon Abrahamsb., 2002** |
| **3** | **Pelargonidin derivatives** | **56.831** | **282**  **527** | **271.2[Pg+H]^+^** |  |
| **4** | **Pelargonidin derivatives** | **57.605** | **284**  **534** | **271.3[Pg+H]^+^** |  |
| **6** | **Kaempferol 7-*O*-rhamnopyranoside** | **21.988** | **257**  **347** | **287.2[Km+H]^+^**  **432.7[M+H]^+^** | **Jin-Ying Gou, Felipe F. Felippes et al., 2011** |
| **7** | **Quercetin 3-*O*-rhamnoside 7-*O*-rhamnoside** | **28.353** | **266**  **347** | **303.0[Qr+H]+**  **449.0[Qr+Rha+H]+** | **Graham., 1998** |
| **8** | **Kaempferol derivatives** | **39.481** | **258**  **351** | **287.3[Km+H]^+^** |  |
| **10** | **Kaempferol 3-*O*-glucoside 7-*O*-rhamnoside.** | **48.352** | **266**  **346** | **287.2[Km+H]^+^**  **433.1[Km+ Rha+H]^+^** | **Takayuki Tohge, Yasutaka Nishiyama et al., 2005** |
| **12** | **Kaempferol 3-*O*-rhamnoside 7-*O*-rhamnoside** | **54.266** | **264**  **341** | **286.9[Km+H]^+^**  **433.0[Km+ Rha+H]^+^** | **Jin-Ying Gou, Felipe F. Felippes et al., 2011** |

There was not obvious aglycone ion corresponded to peak 5, peak 9 and peak 13, thus these three peaks were not used for quantitative analysis.

According to the analysis of standard, we found that anthocyanin has absorbance peak at both 520nm and 360nm, but flavonol only has absorbance peak at 360nm. HPLC results revealed that the retention time of peak 11 is same to peak 2.Therefore, we deduce that peak 11, which is part of the peak 2, is a kind of anthocyanin, and it was not used for the quantitative analysis of flavonol.

**Table B. HPLC-DAD and HPLC-ESI-MS analysis of flavonoid in acidic MeOH-H_2_O extracts of the wild-type petunia and *FhCHS1* over-expressing lines**

| **Peak number** | **Identifacation/tentative identification** | **Retention time (min)** | **λmax (nm)** | **ESI-MS (m/z)** | **References** |
| --- | --- | --- | --- | --- | --- |
| **1** | **Peonidin-rutinoside** | **15.478** | **253**  **511** | **323.1[Pn+Na]+**  **931.1[M+Na]+** |  |
| **2** | **Peonidin 3-glucoside** | **19.995** | **252**  **521** | **301.1[Pn+H]^+^**  **463.5[M+H]^+^** | **Tatsuzawa et al., 1996**  **Toshio Andoa,* Fumi Tatsuzawa et al., 2000** |
| **3** | **Cyanidin 3-rutinoside** | **45.662** | **255**  **532** | **287.2[Cy+H]^+^**  **433.1[Cy+Rha+H]^+^** | **R. J. GRIESBACH, S. ASEN**  **et al., 1991** |
| **4** | **Cyanidin derivatives** | **48.926** | **255**  **538** | **287.1[Cy+H]^+^** |  |
| **5** | **Peonidin derivatives** | **52.489** | **256**  **531** | **301.1[Pn+H]^+^** |  |
| **6** | **Quercetin derivatives** | **4.987** | **250**  **331** | **303.0[Qr+H]^+^** |  |
| **7** | **Quercetin-glucuronide** | **6.217** | **249**  **331** | **302.9[Qr+H]^+^**  **478.9[M+H]^+^** | **MARK O.DOWNEY, JOHN S.HARVEY et al., 2003** |
| **8** | **Kaempferol 3-glucoside** | **9.331** | **266**  **345** | **287.2[Km+H]^+^**  **449.2[M+H]^+^** | **R.J. GRIESBACH and S. ASEN.,1990** |
| **9** | **Kaempferol 7-glucoside** | **13.227** | **249**  **331** | **286.8[Km+H]^+^**  **449.2[M+H]^+^** | **R.J. GRIESBACH and S. ASEN.,1990** |
| **10** | **Kaempferol 3,7-diglucoside** | **45.107** | **265**  **341** | **287.1[Km+H]^+^**  **633.1[M+H]^+^** | **R.J. GRIESBACH and S. ASEN.,1990** |
| **11** | **Kaempferol derivatives** | **49.461** | **265**  **348** | **286.9[Km+H]^+^** |  |

**Reference list for Tables A and B**

**1. Tohge T, Nishiyama Y, Hirai MY, Yano M, Nakajima J, et al. (2005) Functional genomics by integrated analysis of metabolome and transcriptome of *Arabidopsis* plants over-expressing an MYB transcription factor. Plant J 42: 218-235.**

**2. Stephen J. Bloor^a^, Sharon Abrahams^b^ (2002) The structure of the major anthocyanin in *Arabidopsis thaliana*. Phytochemistry 59: 343-346.**

**3. Gou JY, Felippes FF, Liu CJ, Weigel D, Wang JW (2011) Negative regulation of anthocyanin biosynthesis in *Arabidopsis* by a miR156-targeted SPL transcription factor. Plant Cell 23: 1512-1522.**

**4. Graham TL (1998) Flavonoid and flavonol glycoside metabolism in *Arabidopsis*. Plant physiol Biochem 36: 135-144.**

**5. Tatsuzawa F, Saito, N., Yokoi, M., (1996) Anthocyanins in the flowers of *Cymbidium*. Lindleyana 11: 214-219.**

**6. Toshio Ando^a^, Fumi Tatsuzawa^b^, Norio Saito^c^, Motoko Takahashi^a^,, Yuko Tsunashima^a^ HN, Hitoshi Watanabe^a^, Hisashi Kokubun^a^,, Ritsuko Hara^d^ HS, Goro Hashimoto^e^ (2000) Differences in the floral anthocyanin content of red petunias and *Petunia exserta*. Phytochemistry 54: 495-501.**

**7. R. J. GRIESBACH SAaBAL (1991) PETUNIA HYBRIDA ANTHOCYANINS ACYLATED WITH CAFFEIC ACID. Phymchmistry 30: 1729-1731.**

**8. MARK O. DOWNEY^1^, JOHN S.HARVEY^1,3^ and SIMON P. ROBINSON^1,3,4^ (2003) Analysis of tannins in seeds and skins of Shiraz grapes throughout berry development. Australian Journal of Grape and Wine Research 9: 15-27.**

**9. ASEN RJGaS (1990) CHARACTERIZATION OF THE FLAVONOL GLYCOSIDES IN PETUNIA. Plant Science 70: 49-56.**
